# Short Statement of the Result of the Practice in the Hospital for Prisoners of War at Norman Cross

**Published:** 1804-10-01

**Authors:** Leonard Gillespie

**Affiliations:** appointed Physician to the Fleet in the Mediterranean Sea


### Short Statement of the Result of the Practice in the Hospital for Prisoners of War at Norman Cross. *Communicated by* Leonard Gillespie, *appointed Physician to the Fleet in the Mediterranean Sea.*

THE depôt for prisoners of war, established last war on the hill of Norman Cross, is situated on the north road, seventy-six miles north of London, and overlooks on the north-east the lake called Whittlesea Mere, distant about three miles, together with the extensive plains of fen-land, which extend for upwards of thirty miles in a north-east direction from Norman Cross to the borders of the gulph on this coast, called Boston Deeps. The buildings are of wood, and have been occupied by French and Dutch prisoners of war since the commencement of the present war in numbers varying from 1600 to 1000, who have enjoyed a very tolerable state of health since their confinement at this place, as will appear by the enclosed abstract of the numbers received into the hospital. The air of Norman Cross, notwithstanding the vicinity to the fen-lands, is salubrious; the soil is dry and calcareous; the water is drawn from wells in depth from thirty to sixty feet, and is a little hard, being impregnated with a small quantity of earthy salt, probably gypsum, preventing its lathering with soap. The patients in the hospital are, to the honor of the Commissioners for Sick and Wounded Seamen, under whose protection they are placed by Government, treated precisely as our own seamen are in naval hospitals, and of course have every proper attention paid to their comfort, and to the re-establishment of their health. The diseases most prevalent amongst the prisoners of war at Norman Cross, during the winter, were catarrhal complaints, sometimes degenerating into peripneumonia and carditis, fatal instances instances of which occurred, indicated by great anxiety, oppressive pain at the sternum, irregular pulse, and dry cough; and in which cases, dissection proved, that the heart, as well as the lungs, had been really inflamed. Several of these catarrhal complaints also include phthisis pulmonalis.

Rheumatic complaints were also very common, and numbers of the patients were afflicted with rheumatic fever of a remittent type, protracted beyond the period of a lunation.

Many cases of troublesome chilblains occurred to the prisoners who had not sufficient cloathing in the prison; and the itch affected about one hundred of them, who in the spring were without much difficulty cured in the usual manner. During the spring and summer months some hundreds of prisoners having been sent from prison ships in the river Medway, many of them were affected with tertian fevers; in a few cases, of the regular double tertian type; remittent and intermittent fevers also attacked a few of those prisoners who had remained at Norman Cross during the preceding winter.

A few instances of putrid fever occurred in some patients, which, from the symptoms and protracted length of the disease, might be justly termed typhus; whilst in two or three cases the violence of the symptoms, the ardent heat of skin, intense thirst, parched foul tongue, icteritical suffusion of the skin and of the eye on or before the sixth day, atrabiliary or dark alvine evacuations, and rapid progress of the diseases, seemed to entitle it to the appelation of febris ardens of Hippocrates and his disciples, or to that of the yellow fever (vulgarly so called) of the West Indies.

In no one of these cases was the disease propagated by contagion; the common precautions to prevent it were practised. In those cases of ardent fever it was found highly advantageous to shave the head, to sponge the whole of the body frequently in the day with vinegar, to administer saline draughts in the act of effervescence, to give lemonade for drink, and to administer frequent cooling acidulated injections, avoiding the bringing on of a diarrhœa, or when present, moderating it by the infusion of Columbo root aided by rice water, sago, arrow root, gruel, &c. In the low stage of the disease large blisters successively applied, and the discharge from which was kept up for some time, were principally depended on, aided by wine, camphor, and bark. In intermittent fevers the use of of ligatures on the extremities were frequently attended with the effect of checking the violence of the paroxysm, when applied before the cold fit came on.

The scurvy, in its incipient stage, affected many of the prisoners who had been sent from Chatham, and who had lately come from the West India Islands; the usual symptoms of this disease here made their appearance, as, laxity of and bloody gums, looseness of the teeth, fœtid breath, discoloration, a degree of rigidity and contraction in the flexor and adductor muscles of the lower extremities. These patients generally soon recovered by the use of vegetables aided by the fresh citric acid. It is proper to observe, that the patients affected with itch, a slight degree of scurvy, trifling ulcers (which were few in number and of a benign character, evincing the salubrity of the air of the depot) were, together with other patients affected with slight complaints, treated in the prison, which is airy and surrounded by tolerably spacious airing grounds, and consequently were not admitted into the hospital.

*Copy of Abstracts of Quarterly Books containing the Number of Patients received, discharged, cured, dead, &c. in the Hospital for Prisoners of War, at Norman Cross, near Stilton in Huntingdonshire.*

| | | | |
|---|---|---|---|
| 1803. | Last quarter. Received in the quarter ending the 31st of December | | 125 |
| | Discharged cured in ditto | 81 | |
| | Dead in ditto | 3 | |
| | Remaining in the hospital, Dec. 31st. | 41 | |
| | | | 125 |
| 1804. | First quarter. Remaining Dec. 31st. | 41 | |
| | Received in the quarter | 213 | 254 |
| | Discharged cured in ditto | 179 | |
| | Dead in ditto | 3 | |
| | Remaining on the 31st of March | 72 | |
| | | | 254 |
| 1804. | Second quarter. Remaining | 72 | |
| | Received in the quarter | 105 | 177 |
| | Escaped in ditto | 2 | |
| | Dead in ditto | 4 | |
| | Discharged cured in ditto | 111 | |
| | Remaining June the 30th | 60 | |
| | | | 177 |

1804. Third

| | | | |
|---|---|---|---|
| 1804. Third quarter, up to Aug. 31. Remaining | 60 | | |
| Received up to ditto - - - - - - - | 57 | — 117 | |
| Discharged cured up to ditto - - - | 63 | | |
| Dead up to ditto - - - - - - - - | 3 | | |
| Remaining on the 31st of August - - | 51 | | |
| | —— | 117 | |

*Diseases, of which the Patients died.*

| | | | |
|---|---|---|---|
| 1803. | Last quarter. | Phthisis Pulmonalis - - - | 3 |
| 1804. | First quarter. | Peripneumonia - - - - - - | 1 |
| | | Ditto with Carditis - - - - | 2 |
| | Second ditto. | Ardent fever - - - - - - - | 2 |
| | | Typhus fever - - - - - - - | 1 |
| | | Phthisis Pulmonalis - - - | 1 |
| | Third ditto. | Ulceration of the urinary bladder | 1 |
| | | Phthisis Pulmonalis - - - - | 1 |
| | | Typhus - - - - - - - - - | 1 |
| | | Total - | 13 |

